# Clinical and Molecular Characteristics and Outcome of Cystic Partially Differentiated Nephroblastoma and Cystic Nephroma: A Narrative Review of the Literature

**DOI:** 10.3390/cancers13050997

**Published:** 2021-02-27

**Authors:** Sophie E. van Peer, Corine J. H. Pleijte, Ronald R. de Krijger, Marjolijn C. J. Jongmans, Roland P. Kuiper, Marc R. Lilien, Martine van Grotel, Norbert Graf, Marry M. van den Heuvel-Eibrink, Janna A. Hol

**Affiliations:** 1 Princess Máxima Center for Pediatric Oncology, 3584 CS Utrecht, The Netherlands; c.j.h.pleijte@umcutrecht.nl (C.J.H.P.); R.R.dekrijger@umcutrecht.nl (R.R.d.K.); M.C.J.Jongmans-3@umcutrecht.nl (M.C.J.J.); r.kuiper@prinsesmaximacentrum.nl (R.P.K.); m.vangrotel@prinsesmaximacentrum.nl (M.v.G.); M.M.vandenheuvel-eibrink@prinsesmaximacentrum.nl (M.M.v.d.H.-E.); J.hol@prinsesmaximacentrum.nl (J.A.H.); 2 Department of Pathology, University Medical Center Utrecht (UMCU), 3584 CX Utrecht, The Netherlands; 3 Department of Clinical Genetics, University Medical Center Utrecht (UMCU), 3584 CX Utrecht, The Netherlands; 4 Department of Pediatric Nephrology, Wilhelmina Children’s Hospital, University Medical Center Utrecht, 3584 CX Utrecht, The Netherlands; M.Lilien@umcutrecht.nl; 5 Department of Pediatric Oncology & Hematology, Saarland University Medical Center and Saarland University Faculty of Medicine, D-66421 Homburg, Germany; Norbert.Graf@uks.eu

**Keywords:** cystic partially differentiated nephroblastoma, cystic nephroma, CPDN, CN, pediatric, renal tumor

## Abstract

**Simple Summary:**

Although renal tumors in children are mostly solid masses, cystic renal tumors also occur. The most likely diagnoses for cystic renal tumors include cystic partially differentiated nephroblastoma and cystic nephroma. Since these tumors are rare, limited information on the treatment, clinical and molecular characteristics, and outcome is available. In this review, we aim to summarize all reported patients with cystic partially differentiated nephroblastoma and cystic nephroma. We identified 113 cystic partially differentiated nephroblastoma and 167 cystic nephroma patients. Surgery was the cornerstone of treatment for both tumor types and chemotherapy was generally not recommended. Cystic nephroma was often related to *DICER1*-mutations and second tumors, whereas cystic partially differentiated nephroblastoma was related to somatic hyperdiploidy, although testing was rare. The outcome for both tumors is favorable. This study provides information for treatment decisions and stresses the importance of a central review of radiology and pathology, as well as referral to a clinical geneticist.

**Abstract:**

In children presenting with a predominantly cystic renal tumor, the most likely diagnoses include cystic partially differentiated nephroblastoma (CPDN) and cystic nephroma (CN). Both entities are rare and limited information on the clinical and molecular characteristics, treatment, and outcome is available since large cohort studies are lacking. We performed an extensive literature review, in which we identified 113 CPDN and 167 CN. The median age at presentation for CPDN and CN was 12 months (range: 3 weeks–4 years) and 16 months (prenatal diagnosis–16 years), respectively. No patients presented with metastatic disease. Bilateral disease occurred in both entities. Surgery was the main treatment for both. Two/113 CPDN patients and 26/167 CN patients had previous, concomitant, or subsequent other tumors. Unlike CPDN, CN was strongly associated with somatic (*n* = 27/29) and germline (*n* = 12/12) *DICER1*-mutations. Four CPDN patients and one CN patient relapsed. Death was reported in six/103 patients with CPDN and six/118 CN patients, none directly due to disease. In conclusion, children with CPDN and CN are young, do not present with metastases, and have an excellent outcome. Awareness of concomitant or subsequent tumors and genetic testing is important. International registration of cystic renal tumor cohorts is required to enable a better understanding of clinical and genetic characteristics.

## 1. Introduction

Among children with renal tumors, cystic partially differentiated nephroblastoma (CPDN) and cystic nephroma (CN) are rare diagnoses. CPDN and CN present as grossly or entirely multicystic (multilocular) renal masses, while other pediatric renal tumors are usually visible as solid masses on imaging studies, and 80–90% represent Wilms tumors (nephroblastomas) [1].

Establishing the correct diagnosis requires careful histological examination of the resected tumor, since imaging studies fail to discriminate between the two entities [2]. Histologically, CPDN is characterized by the presence of undifferentiated (blastemal) cells in the septa of the cysts, and by the lack of solid components (tumor “nodules”). By definition, CN does not contain any solid components or blastemal cells in the septa. Only mature epithelial tubules, possibly from pre-existent normal renal tissue, are acceptable for the diagnosis. Wilms tumors may contain cysts, particularly after pre-operative chemotherapy, but the presence of solid tumor components or nodules excludes a diagnosis of CPDN or CN [3]. CN has been found to be strongly associated with mutations in the *DICER1* gene (both germline and somatic) [4], which is not the case for CPDN, and whether there is a biological relationship between the two entities remains to be clarified. Currently, treatment recommendations are based on limited evidence, and a comprehensive overview of the clinical characteristics, molecular aberrations, role of different treatment components, and outcomes of CPDN and CN is not available. Here, we present the results of an extensive literature review in which we aim to characterize the clinical and biological features and outcome of CPDN and CN.

## 2. Methods

### 2.1. Literature Search

A complete search of the PubMed and EMBASE databases was performed to identify all reports describing pediatric patients (age 0–18 years) with CPDN and/or CN published until June 2020. We also included articles that described cystic Wilms tumors (cWT), as this is the most important differential diagnosis in children with CPDN and CN. Synonyms for CPDN, CN, and cWT were used as search terms (full search terms are presented in Table S1). All manuscripts were screened by two independent reviewers (S.E.v.P. and J.A.H.). To be eligible for inclusion, a study had to report well-described patients with CPDN or CN aged 18 years or younger; had to be an original article; had to be written in English, German, Spanish, or Dutch language; and had to be available as a full text. A cross reference check was used to identify potential additional reports. In the case of multiple case reports from the same authors or institution, we checked for previously reported patients based on clinical descriptions and excluded duplicate cases if applicable. As cWT is not a separate entity in the renal tumor histology classification by the International Society of Pediatric Oncology Renal Tumor Study Group (SIOP-RTSG), we focused on CPDN and CN in this review.

### 2.2. Classification of Tumors

Where necessary, tumors in this literature review were (re-)classified as CPDN or CN by the authors and a pathology review member of the SIOP-RTSG based on pathology data provided in the reports using the SIOP staging criteria for renal tumors of childhood [5]. This (re-)classification was performed for three reports, as two were published before 1989 [6,7], when the first diagnostic criteria by Joshi and Beckwith were established, or when reports were not in agreement with the current definition of CPDN and CN (*n* = 1) [2], which was revised in 2002 [5,8] (Table S2).

## 3. Results

**Figure 1:**
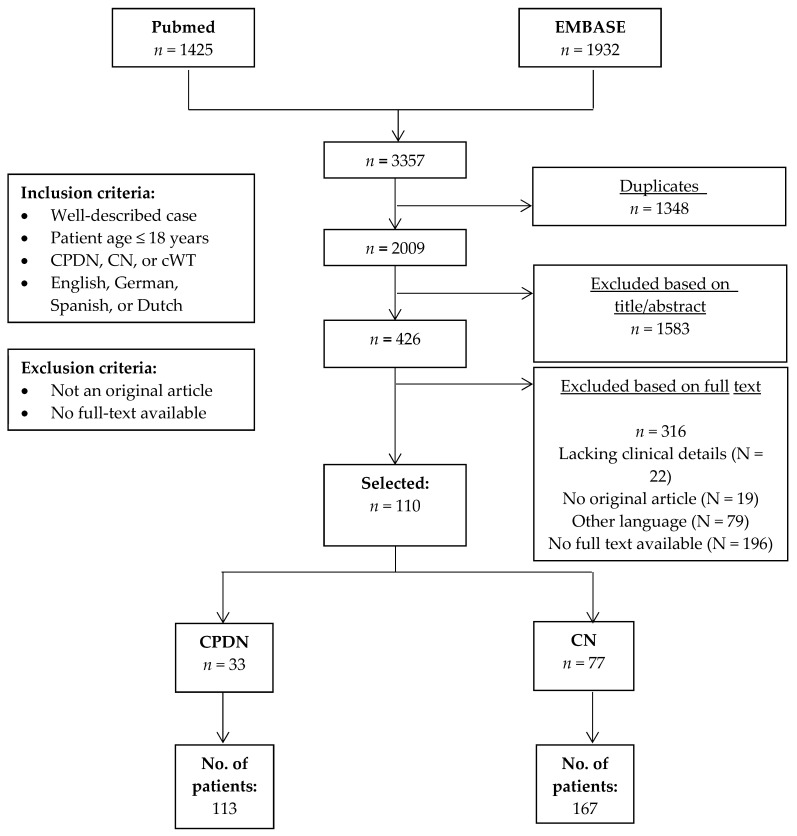
Flowchart of the literature search (updated 15 June 2020). *n* = number of reports. CN: cystic nephroma; CPDN: cystic partially differentiated nephroblastoma; cWT: cystic Wilms tumor.

After the removal of duplicates, the literature search yielded 2009 articles, of which 110 were included after title/abstract and full-text screening (Figure 1). From these 110 reports, a total of 280 patients were identified (167 patients with CN and 113 patients with CPDN) (Table 1). Three additional CPDN cases were identified in our literature search, in which detailed genetic characteristics of the tumor were investigated, but yielded no clinical data [9,10,11]. These three cases are therefore not included in the description of clinical characteristics; the molecular findings of these tumors are included in the table describing the molecular characteristics of CPDN.

### 3.1. Cystic Partially Differentiated Nephroblastoma (CPDN)

#### 3.1.1. Clinical Characteristics and Disease Stage

Of the 113 identified patients with CPDN (Table 1 and Table S3) with available clinical data, 73 were male and 38 were female (sex was not specified in the remaining two). The median age at diagnosis was 12 months (*n* = 106, range: 3 weeks–4 years). Clinical presentation was described in detail in 44 patients (Table S4), with the most common presentation being a palpable abdominal mass (*n* = 40/44). The median tumor weight at surgery was 484 g (range 170–3110 g, *n* = 44) and the median described tumor volume was 338 mL (range 122–405 mL, *n* = 12).

A total of 108 patients presented with unilateral disease, while five presented with bilateral disease (4.4%) [8,12,13,14,15], and none of the patients had metastatic disease. Three of the bilateral cases had CPDN in both kidneys [12,14,15]. Of the other two patients, one had CPDN with contralateral nephroblastomatosis [13] and one had CPDN with Wilms tumor, with focal anaplasia in the other kidney [8]. The SIOP abdominal stage, according to the SIOP staging criteria [16], could be identified in or assessed by the authors (based on the information provided in the reports) for 72 patients. Stage I was recorded for 62 patients, stage II for one patient with tumor extension beyond the kidney [12], and stage III for nine patients. Stage III disease was based on invasion of the abdominal wall (*n* = 1) [17], pre- or intra-operative tumor rupture (*n* = 7) [8,12,18,19], or not specified [14]. In six patients, the tumor prolapsed into the ureter. Information on lymph node involvement was documented for 52 patients and was negative in all cases.

#### 3.1.2. Treatment and Outcome

Pre-operative chemotherapy administration was reported in 20 out of 98 patients with reported details on the pre-operative therapy and response (Table 2) [12,14,15,17,19,20,21,22,23,24], including two of the five patients with bilateral disease [12,14]. None of the pre-operatively treated patients showed response to chemotherapy on imaging or histological examination. In three of them, pre-operative imaging revealed an increase in tumor size after chemotherapy [14,15,23]. Seventy-eight patients underwent upfront surgery without chemotherapy. Surgical resection (primary or after chemotherapy) of the tumor was performed in 112/113 patients. The only patient who did not have surgery was a patient with extensive comorbidity, where a conservative treatment approach was chosen, following the parents’ wishes [25]. The surgical approach was radical nephrectomy in 94 cases and nephron sparing surgery (NSS) in eight patients, with the latter including all five bilateral patients [8,12,13,14,15]. The type of surgery was not described in 10 cases.

Post-operative chemotherapy administration was reported in 47 patients. Fifty-two patients did not receive any post-operative therapy based on the histological diagnosis of CPDN, while information on post-operative therapy was not available in 14 patients. Reported reasons for post-operative treatment (if specified) included tumor rupture or spill (*n* = 7) [8,12,18,19], the presence of nephroblastomatosis (*n* = 1) [13], and the aim to reduce serum α-fetoprotein levels, which occurred in one patient [21]. In the other 38/47 post-operatively treated patients, reasons for this decision were not specified. Pre-operative chemotherapy strategies were vincristine, actinomycin-D, and doxorubicin in four patients; vincristine and actinomycin-D in one patient; actinomycin-D alone in one patient; and not specified in 14 patients. Post-operative treatment strategies were vincristine and actinomycin-D in 30 patients; vincristine alone in two patients; vincristine, actinomycin-D, and doxorubicin in one patient; and not specified in 14 patients.

Only four case reports described detailed treatment-related toxicity occurring in a total of seven patients [12,17,18,22]. Two of these seven patients suffered from vincristine-related peripheral neuropathy [12], two developed intermittent viral illnesses and fevers [18,22], and three developed hematologic toxicity, of which one had recurrent episodes of sepsis [12,17,22]. These toxicities resulted in dose reduction or a delay of chemotherapy in these patients and one patient required supportive care therapy consisting of blood and platelet infusions [17]. A total of four relapses were described (Table 3) [8,15,18]. In three of these cases, a differential diagnosis of cystic Wilms tumor could not be excluded based on the histological description in the original report, as most likely histology was not reviewed by a panel of pediatric pathologists involved in the SIOP-RTSG or Children’s Oncology Group (COG), and a detailed description was lacking. Molecular studies were not performed for any of the patients that relapsed. A total of six deaths were described, including two of the four relapsed cases, in whom cause of death was unrelated to CPDN or unknown [15]. Two of the deaths included a patient with a concomitant stage III Wilms tumor with focal anaplasia [8] and a patient with an underlying genetic syndrome (mosaic variegated aneuploidy), which will be discussed below [25]. The last two patients with CPDN who died were reported in a low income country and the cause of death was not possible to retrieve [19]. We did not identify any relationship between the outcome and chemotherapy treatment in these patients and large series of data that allow statistical analysis are not available.

#### 3.1.3. Molecular Testing and Genetic Predisposition

Seven of the 113 CPDNs were tested for somatic *DICER1* gene mutations, and all showed negative results (Table 4). Eight CPDN tumors had been karyotyped, of which seven revealed hyperdiploidy (varying from 50 to 52 chromosomes in tumor cells) [10,11,25,27,28,29]. In one case of CPDN, somatic loss of heterozygosity for 11p13, 11p15, and 16q was found in the CPDN, as well as in the contralateral nephrogenic rest (all detected by PCR) [10] (Table 4). *WT1* mutation analysis was not performed for any of the CPDNs.

Genetic predisposition was reported in two cases. A 7-month-old female was diagnosed with Mulibrey Nanism caused by a germline *TRIM37* mutation [30]. Another patient was described with mosaic variegated aneuploidy characterized by premature centromere divisions and mosaic hyperdiploidy in both tumor and healthy cells. This patient also had a rhabdomyosarcoma and several congenital anomalies, which were both previously described in patients with mosaic variegated aneuploidy [31]. No germline genetic testing for the causative mutation was performed for this patient [25].

**Table 4:** Molecular testing and genetic predisposition in tested patients with CPDN.

Reference	No. of Tested Patients	Karyotype	*DICER1* Mutation		Other molecular/Germline Aberrations
			Somatic	Germline	
Charles et al. [10]	1	52, XY, +2, +5, +7, +8, +17, +18. LOH p13b, p15T, and 16q in CPDN and nephrogenic rest (somatic)	NA	NA	-
Cummings et al. [9]	1	46XY, no LOH 11p13, 11p15, 16q, or 7p (somatic)	NA	NA	-
De Chadarévian et al. [27]	1	51XY, +7, +8, +12, +13, +17, one cell disomic for chromosome 7 (somatic)	NA	NA	-
Doros et al. [32]	6	Not performed	0/6	0/6	-
Kaneko et al. [29]	2	Case no. 1 (74 in article): 50, XY, +6, +7 + 12, + 13, + 18/51, + 17 Case no. 2 (384 in article): 50, X, -Y, +6, +12, +13, +der(?)t(1;)(q21;?), +der(?)t(1;?)(q21;?) (both somatic)	NA	NA	-
MdZin et al. [11]	1	Hyperdiploidy with presence of trisomy 12 (somatic)	NA	NA	-
Stout et al. [14]	1	Not performed	0/1	0/1	-
Timmons et al. [28]	1	Clonal hyperdiploid karyotype, mostly: 51, XY, +8, + 12, +17, + 19, +20. trisomy 8 was present in every hyperdiploid examined. Of the three hyperdiploid cells with 50 chromosomes, two lacked the third copy of chromosome 17 and one lacked the third copy of chromosome 12 (somatic)	NA	NA	-
Furukawa et al. [25]	1	Clonal hyperdiploid karyotype, mosaic trisomies occurring in 18 to 23% of somatic cells, in nephroblastoma cells mostly: 51, XX, +2, +7, +8, +10, +12. Trisomy 11, 18, 19, and 20 and monosomy 17 were also present in some aneuoploid cells (somatic and germline)	NA	NA	Mosaic variegated aneuploidy
Taskinen et al. [30]	1	Not performed	NA	NA	Mulibrey Nanism (germline *TRIM37* mutation)
TOTAL	16	TOTAL	0	0	

CPDN: cystic partially differentiated nephroblastoma; LOH: loss of heterozygosity; NA: not available.

For the other patients, germline genetic testing of *DICER1* or other genes was not described, and they did not have additional tumors. One exception was the patient with bilateral disease who had a Wilms tumor with focal anaplasia in the contralateral kidney (no information on molecular or germline testing available). For the remaining four patients with bilateral disease, germline testing was not described.

### 3.2. Cystic Nephroma (CN)

#### 3.2.1. Clinical Characteristics and Disease Stage

Of the 167 identified patients with CN (Table 1 and Table S5), 93 were male and 72 were female (for two patients, sex was not specified). The median age at diagnosis was 16 months (*n* = 113, range prenatal diagnosis—16 years). Similar to CPDN, a palpable abdominal mass was, when documented, the most frequently reported presenting symptom (62/88, 70%) (Table S4). The median reported tumor weight at surgery was 540 g (range 120–4000 g, *n* = 60) and the median tumor volume was 292 mL (range 6.7–1490, *n* = 37). Unilateral disease was reported in 158 patients and nine patients had bilateral CNs (5.4%), including one patient with metachronous tumors [33]. None of the patients had metastatic disease. The SIOP local stage was reported or assessed by the authors according to the SIOP staging criteria [16] in 52 patients, of which 51 patients had stage I and one had stage III disease based on intraoperative tumor rupture [3]. In three patients, the tumor prolapsed into the ureter. Information on lymph node involvement was documented for 24 patients and none were positive for tumor cells.

#### 3.2.2. Treatment and Outcome

Information on pre-operative chemotherapy administration was available for 142/167 patients (Table 2). Nine patients were treated with upfront chemotherapy [2,3,7,20,34,35,36,37]. The response was only reported in one chemotherapy-treated patient (with bilateral CN) [32]. In the other eight chemotherapy-treated patients, the response was not described on imaging or histological examination. Pre-operative radiotherapy was administered in five patients, all in reports from before 1994 [7,20,37,38]. In three of these patients, a pre-operative suspicion of a Wilms tumor was reported [7,37,38], for which pre-operative radiotherapy was considered standard at that time (prior to 1980). In two other cases, the treatment choice was not explained [20]. Surgery (upfront or after chemotherapy) was performed in 165/167 patients.

The surgical approaches included radical nephrectomy in 121 cases and NSS in 28 cases, while the type of surgery was not available for 16 patients. Among the two patients who did not have surgery, one patient had prenatally been diagnosed with CN and polyhydramnios and this patient died before birth due to the premature rupture of membranes at the gestational age of 16 weeks and six days [39]. The other patient had pre-operatively been diagnosed with CN through a biopsy, leading to a conservative approach, after which the tumor remained unchanged during a follow-up of eight months [35].

Post-operative chemotherapy administration was reported in five patients [40,41,42,43,44]. Details on response were not available for these post-operatively-treated patients. Fifty-two patients did not receive post-operative chemotherapy and information on post-operative therapy was not available for 110 patients. Reported reasons for chemotherapy treatment after nephrectomy were concomitant pleuropulmonary blastoma (PPB) (*n* = 1) [40], rapid pre-operative growth of the CN (*n* = 1) [41], suspicion of a Wilms tumor before obtaining histology results (*n* = 1) [44], or not specified (*n* = 2) [42,43]. Pre-operative chemotherapy strategies were vincristine and actinomycin-D in five patients; ifosfamide, carboplatin, and etoposide in one patient; and not specified in three patients. Post-operative chemotherapy strategies were vincristine and actinomycin-D in two patients; vincristine alone in one patient; a combination of vincristine, doxorubicin, cyclophosphamide, iphosphamide, carboplatin, and etoposide in one patient (PPB treatment); and not specified in one patient. Three reports described treatment-related toxicity; two patients developed post-operative sepsis (no chemotherapy) and died [38,45], and one patient suffered from chemotherapy-related neutropenia [44].

One local relapse was described, most likely due to an incomplete resection of the prior tumor (Table 3) [26]. Six patients died during follow up; in two cases related to CN treatment (post-operative sepsis) [38,45], and in four cases due to complications caused by treating concomitant disease including PPB (*n* = 2) [46,47] and brain hemorrhage during surgery for cerebral spindle cell sarcoma (*n* = 1) [48]. In one patient, intrauterine death was reported [39]. We did not identify any relationship between the outcome and chemotherapy treatment in these patients and large series of data that allow statistical analysis are not available.

#### 3.2.3. Molecular Testing and Genetic Predisposition

*DICER1* mutations (germline and/or somatic) were identified in 33/35 tested cases (Table 5). This included 12 patients with confirmed germline *DICER1* mutations (or 14q32 deletion in one case); six of them also had a somatic *DICER1* mutation in the tumor [48,49,50,51,52,53]. This was not assessed or described in the remaining five patients [54,55,56]. In 21 patients, *DICER1* mutations were identified in the tumor, but germline DNA was not analyzed. Among them, 16 tumors harbored both a missense and truncating mutation, the latter of which possibly represents germline events, as suggested by the study of Doros et al. [32].

Among 12 CN patients with germline *DICER1* mutations, ten had previously or subsequently developed other tumors in addition to CN (Table 6). In the remaining 155 patients, including nine patients with bilateral CN, germline genetic testing (*DICER1* or other testing) was not described. Prior to or following CN diagnosis, a total of 26 patients (for 17 of whom, the germline *DICER1* status was not documented) developed *DICER1*-related tumors (e.g., PPB, multinodular goiter, embryonal rhabdomyosarcoma, malignant teratoid ciliary body medulloepithelioma, and others). Of the 26 CN patients that developed other tumors, four developed these tumors before the CN, 14 were concomitant, and eight patients developed the other tumors after the CN. The time range between the tumors varied from 1 month to 4 years. Two patients presented with a concomitant contra- or ipsilateral Wilms tumor.

### 3.3. Differential Diagnosis of CN and CPDN

During our literature review, we encountered a broad range of other diseases that are included in the differential diagnosis of cystic renal lesions. Reported non-malignant cystic renal diseases included simple cysts, autosomal dominant or recessive polycystic kidney disease, medullary cystic kidney disease, nephronophthisis, multicystic kidney disease, renal cysts and diabetes syndrome/HNF1B-associated renal disease and a medullary sponge kidney. Malignant diseases included cystic subtypes of renal cell carcinoma and cWT [64]. Since cWT, CPDN, and CN were often proposed to be part of a spectrum, we included cWT in our literature search. We identified 18 cases of cWT, many of which were reported > 20 years ago, which are summarized in Table S6. The median age was 10 months (individual ages available from 14 patients) and presenting symptoms were most frequently an abdominal mass and, more rarely, fever or malaise. Two patients presented with metastatic disease. Except for two patients (siblings) with an unspecified underlying syndrome, all patients were successfully treated with surgery and chemotherapy, or surgery alone. In this review, we focused on CPDN and CN, as cWT is not a separate entity in the SIOP-RTSG classification, and after our search, we noticed that cWT reports, apart from being > 20 years old, were scarce.

## 4. Discussion

This literature review, in which we identified 113 patients with CPDN and 167 patients with CN, demonstrated that CPDN and CN are clinically difficult to distinguish from each other. Both tumors are characterized by a similar younger age at diagnosis (median age of 12 and 16 months, respectively) than other renal tumors, such as a Wilms tumor (median three years) or renal cell carcinoma (adults and adolescents) [65]. Most patients present with a palpable or visible abdominal mass, similar to what is observed in patients with Wilms tumors. Both CPDN and CN are apparently associated with the absence of lymph node involvement or metastatic disease. They have a very low relapse rate and an excellent outcome. Nevertheless, second primary (malignant) tumors can occur, particularly in patients with CN and underlying DICER1 syndrome, which illustrates the need for awareness and surveillance of these children, even though surgery is the most important treatment modality and response to chemotherapy seems to be limited.

It is challenging to correctly diagnose CPDN or CN based on clinical characteristics or radiology. Imaging studies do not seem to be able to reliably discriminate CN from CPDN or other cystic (Wilms) tumors, although Bosniak classification might help to distinguish these entities from other cystic kidney diseases, such as polycystic kidneys, by the stratification of renal cysts into different subcategories based on imaging features [64]. Biopsies to confirm the diagnosis are not encouraged, as cystic tumors carry a high risk of tumor spillage and the biopsied region may not be representative of the tumor [16]. In children with an entirely cystic (unilateral) tumor on imaging studies, immediate surgery is recommended in most renal tumor protocols, including the recently launched SIOP-RTSG UMBRELLA protocol, albeit after a review by a pediatric radiologist with specific expertise in this field [3]. It is crucial to exclude benign forms of cystic kidney disease as unilateral nephrectomy may accelerate the course of disease in these cases [66]. Chemotherapy and radiotherapy are generally not recommended for CN or CPDN. Nevertheless, we found that pre- and/or post-operative chemotherapy was sometimes administered in the past to patients with CPDN and occasionally to patients with CN. A response to preoperative chemotherapy has not been observed, except for one case of bilateral rapidly growing CN, in which pre-operative chemotherapy administration led to growth arrest, averting end-stage renal disease [32]. Large cohort studies comparing outcomes with and without chemotherapy are not available. It remains questionable whether there is a role for post-operative chemo- and radiotherapy in the case of tumor rupture when tumor spillage may have occurred.

Bilateral disease occurs in both CN (5.3% of patients) and CPDN (4.4% of patients), suggesting a genetic predisposition, or a shared precursor lesion early in kidney development, in these patients. For CN, the occurrence of bilateral tumors is likely largely explained by underlying germline *DICER1* mutations, although only 12/167 reported patients with CN were tested for *DICER1* syndrome. For CPDN, only two patients were reported to have a genetic predisposition, but the majority were not referred for assessment by a clinical geneticist and/or genetic testing and genetic predisposition syndromes may have been missed. Therefore, referral to a clinical geneticist is important for all patients with CN, who should be evaluated for *DICER1* syndrome so that they can receive adequate follow-up, and for patients with CPDN, where genetic testing can be considered based on the presence of bilateral disease, family history, and/or additional phenotypic features.

Both CN and CPDN have been reported to occur in combination with contralateral Wilms tumor. Since the clinical and radiological features of both entities are similar, previous studies have suggested that solid Wilms tumors, CPDN, and CN represent a spectrum of differentiation, with solid Wilms tumors being the least differentiated and CN being the most differentiated end of the spectrum [8]. Alternatively, it has been suggested that CN, CPDN, and Wilms tumors may share a common origin in intralobar nephrogenic rests [36]. The high frequency of *DICER1* mutations in CN and the absence of *DICER1* mutations in CPDN have led Cajaiba et al. to classify CN as a separate entity, rather than as part of a spectrum together with CPDN [4]. It is intriguing that *DICER1* mutations (both germline and somatic) have also been identified in solid Wilms tumor cases [67] and the co-occurrence of CN and a Wilms tumor has been reported twice [36,38]. Somatic *DICER1* mutations have never been identified in CPDN, for which molecular testing has only been sporadically reported [14,32]. Somatic hyperdiploidy appeared to be common in CPDNs of nonsyndromic patients (seven of eight karyotyped cases). Hyperdiploidy has previously also been associated with Wilms tumors, in which the level of hyperdiploidy shows a correlation with the histological subtype [68]. We did not identify any reports describing hyperdiploidy in CN, where karyotyping was only rarely performed. In the future, standardized broad genomic screening may identify aberrations that help to discriminate between cWT, CN, and CPDN, which may even be feasible before surgery by using circulating cell-free DNA techniques [69].

Although CN and CPDN are both considered relatively benign or low-risk lesions, relapses were occasionally reported. In CPDN, four relapses were reported, but the initial histological diagnosis was not always entirely clear in the original report, and we hypothesize that, in some of these cases, solid components may have been present in the cyst walls, suggesting an initial diagnosis of cWT rather than CPDN [8,15,18]. In CN, one local relapse was reported, which was attributed to incomplete resection by the authors of the original report [26]. Deaths in this literature review were due to the toxicity of chemotherapy or comorbidity in both CPDN and CN, rather than disease progression. Notably, the malignant transformation of CN to anaplastic sarcoma [52] or anaplastic malignant mesenchymoma [44] has been reported. A limitation of our review is that the follow-up time was frequently not reported or highly variable between different reports.

## 5. Conclusions

This review summarizes the clinical and molecular characteristics and outcome of all reported CN and CPDN in children. It emphasizes that CN and CPDN are difficult to distinguish based on patient characteristics, presenting symptoms, and radiology. They have an overall favorable outcome and the benefit of chemotherapy seems to be limited. Awareness of concomitant tumors in the kidneys, lungs, and other organs is important, especially in CN patients, and genetic testing and counselling is advised, as well as a central review of histology and radiology, in order to avoid unnecessary treatment and to identify other tumors at an early stage. In the future, genomic sequencing studies (germline and somatic) and the prospective registration of genetic, clinical, and tumor characteristics will likely provide more insight into the biological relationships between CN, CPDN, and Wilms tumor subtypes. International collaboration is needed to prospectively register large cohorts. The SIOP 2016 UMBRELLA protocol, which aims to capture all renal tumors prospectively with enhanced characterization of phenotype and genomic sequencing, and a central review of histology and radiology of the tumors, may identify further unique and discriminating features in the future.

## Figures and Tables

**Table 1:** Clinical characteristics and outcome of cystic partially differentiated nephroblastoma (CPDN) and cystic nephroma (CN).

Tumor Type	No. of Patients	Sex (M/F/NA)	Median Age (Months)	Median Tumor Weight (Grams)	Median Tumor Volume (mL)	Stage						LN + Patients/Tested Patients	Relapse	Survival
						I	II	III	IV	V	NA			
CPDN	113	73/38/2	12 (0.1–36)	484 (170–3110)	338 (122–405)	62	1	9	0	5	37	0/52	4	107
CN	167	93/72/2	16 (0–198)	540 (240–4000)	292 (6.7–1490)	51	0	1	0	9	106	0/24	1	161

M: male; F: female; NA: not available; LN: lymph nodes; +: positive.

**Table 2:** Summary of described therapy of cystic renal tumor patients.

Tumor Type	No. of Patients	Pre-Op CT	Pre-Op RT	Surgery Type				Post-Op Treatment				
				TN	NSS	NA	None	CT	RT	CT + RT	NA	No Post-Op Therapy
CPDN	113	20	0	94	9	9	1	40	0	7	15	52
CN	167	9	5	121	28	16	2	5	0	0	110	52

CPDN: cystic partially differentiated nephroblastoma; CN: cystic nephroma; Pre-op: pre-operative; CT: chemotherapy; RT: radiotherapy; TN: tumor nephrectomy; NSS: nephron-sparing surgery; NA: not available; post-op: post-operative.

**Table 3:** Clinical and treatment characteristics of relapsed patients.

#	Type	Age	Sex	Preop Tx	Initial Histology	Surgery	Postop Tx	Time to Relapse	Histology of Relapse	Relapse Treatment	Survival
1	CPDN [8] ^#^	4m	M	None	>50% of the tissue in the septa contained immature elements. Possibly positive margins.	TN, possibly incomplete	None	1 y/2 y ***	NA	Surgery	Yes
2	CPDN [18] ^#^	1y	M	None	Pre- and intraoperative cyst rupture, cystic tumor, septa contained tissue reminiscent of triphasic WT with nodules of blastema	TN, intraoperative tumor rupture	Chemo: Vincristine and actinomycin-D	6 weeks (while on chemo)	WT	Chemo, surgery	Yes
3	CPDN bilateral [15] ^#^	9m	M	None	NA	Bilateral NSS	None	6 months	Bilateral WT	Chemo, surgery	No *
4	CPDN [15] ^#^	3y	M	Chemo	NA	TN (positive margins)	Chemo	5 months	NA	Chemo	No **
5	CN [26]	6m	F	None	Probable positive margins	NSS (probably positive margins)	None	3 months	NA	NA	Yes

CPDN: cystic partially differentiated nephroblastoma; ^#^: these cases were previously reviewed by Kurian et al., 2018 [15]; preop Tx: pre-operative therapy; postop Tx: post-operative therapy; m: months; y: years; TN: tumor nephrectomy; NSS: nephron sparing surgery; NA: not available; *: death unrelated to CPDN; **: lost to follow up, presumed death by authors; ***: two relapses, chemo: chemotherapy; WT: Wilms tumor.

**Table 5:** Somatic and germline aberrations in tested CN patients.

Reference	No. of Tested Patients	Karyotype (Tumor; Somatic)	*DICER1*-Mutation Positive	
			Tumor	Germline
Saskin et al., 2017 [49]	1	Not performed	c.5113G > A, p.E1705K	Heterozygous c.4566_4570dupCTTTG p.V1524Afs * 38
Fernández-Martinez et al., 2017 [50]	1	Not performed	c.5425G > A (p.G1809R)	Heterozygous c.5387C > T; p.Q1783 *
De Kock et al., 2015 [51]	1	Not performed	c.5439G > T p.(Glu1813Asp)	Heterozygous c.1196_1197dupAG p.(Trp400Serfs * 59)
De Kock et al., 2018 [48]	1	Normal (46, XY)	c.5425G > A, p.G1809R	Heterozygous 5.82 Mb deletion at the 14q32.13q32.2 region
Bardón-Cancho et al., 2017 [57]	1	Not performed	Not tested	Heterozygous c.3540C > G; p.Y1180
Cajaiba et al., 2016 [4]	16	Not performed	12 patients with LOF (nonsense or frameshift) and hotspot mutations 3 patients with hotspot mutations only	Not tested *
Faure et al., 2016 [54]	2	Not performed	Not tested	Patient 1 LOF mutation Patient 2 mutation (NOS)
Li et al., 2017 [58]	7	Not performed	Patient 1. c.5125G > A; p.D1709N Patient 2. c.5437G > A; pE1813K and c.3452_3453delTG; p.V1151Efs12 * Patient 3. c.5425G > A; G1809R Patient 4. c. 5125G > A; p.D1709N and c.958A>T; p.K320 *; Patient 5. c.5428G > C; p.D1810H and c. 3091C > T; p.Q1031 * Patient 6: c.5125G > A; p.D1709N and c.1177C > T; p.Q393 *	Not tested *
Bueno et al., 2017 [55]	2	Not performed	Not tested	Patient 1: Germline *DICER1* variant (NOS) Patient 2: Germline *DICER1* variant (NOS)
Wu et al., 2016 [52]	1	Not performed	c.5438A > G; p.E1813G	Heterozygous c.2450delC; p.P817LfsX15
Apellaniz-Ruiz et al., 2020 [53]	1	Not performed	c.5428G > T, p.D1810Y	c.4308_4311del, p.A1436fs
Dural et al., 2020 [56]	1	Not performed	Not tested	Heterozygous c.1525C>T p.Arg509 *
TOTAL	35	TOTAL	27	12

* These tumors harbored both a missense and a truncating *DICER1* mutation. Germline DNA was not assessed, and the majority of truncating *DICER1* mutations are suspected to be germline mutations; NOS: not otherwise specified; LOF: loss-of-function.

**Table 6:** Other tumors identified in patients with CN and CPDN.

Patient	Age (Months)	Gender	Tumor	*DICER1*		Survival
				Somatic	Germline	
CN (bilateral) [15]	4	Male	ERMS urethra ^###^	NA	NA	Yes
CN [49]	14	Female	NBL *^,##^	+	+	Yes
CN [50]	11	Female	PPB ^###^	+	+	Yes
CN (bilateral) [59]	30	Male	PPB ^##^	NA	NA	NA
CN (bilateral) [60]	9	Male	PPB **^,##^	NA	NA	Yes
CN [30]	18	Female	PPB ^##^	NA	NA	Yes
CN [46]	20	Male	PPB ^###^	NA	NA	No
CN [51]	144	Female	ERMS ovary *^,^**^,#^ Focal nodular liver hyperplasia ^#^, Fibro-adenoma of the breast ^###^	+	+	NA
CN [47]	54	Female	PPB ^###^	NA	NA	No
CN [48]	12	Male	Malignant teratoid CBME, High-grade cerebral SCS, PPB ^###^	Somatic *DICER1* mutations in the CBME and spindle-cell sarcoma	+	No
CN [61]	30	Male	NBL ^##^	NA	NA	Yes
CN [35]	22	Male	PPB ^##^	NA	NA	Yes
CN [40]	32	Male	PPB ^##^	NA	NA	Yes
CN [54]	66	Female	SLCT ^#^	-	+	NA
CN [38]	24	Female	WT ^##^	NA	NA	Yes
CN [62]	5	Female	PPB ^##^	NA	NA	NA
CN [63]	11	Female	PPB	NA	NA	Yes
CN (bilateral) [63]	10	Female	PPB **	NA	NA	Yes
CN [63]	26	Female	PPB	NA	NA	Yes
CN [55]	13	Male	Pineal cyst	-	+	NA
CN [55]	78	Female	ERMS ovary, fibroadenomas, thyroid cysts, exostoses	-	+	NA
CN [36]	21	Female	WT ^##^	NA	NA	Yes
CN [52]	7	Female	ASK ^##^	+ (in CN and ASK)	+	Yes
CN [53]	10	Male	Paratesticular sarcoma ^##^	+ (in CN and paratesticular sarcoma)	+	Yes
CN [56]	12	Female	ERMS uterus ^###^	-	+	Yes
CN [44]	26	Female	Anaplastic malignant mesenchymoma of the kidney ^###^	NA	NA	Yes
CPDN [8]	30	Female	WT ^##^	NA		Yes
CPDN [25]	2	Female	RMS ^#^	Multiple variegated aneuploidy		No

CN: cystic nephroma; CPDN: cystic partially differentiated nephroblastoma; NA: not available; ERMS: embryonal rhabdomyosarcoma; ^#^: prior tumor; ^##^: concomitant tumor; ^###^: subsequent tumor; NBL: neuroblastoma; *: nodular hyperplasia of the thyroid; PPB: pleuropulmonary blastoma; **: small bowel polyp; CBME: ciliary body medulloepithelioma; SCS: spindle cell sarcoma; SCLT: Sertoli-Leydig Cell tumor; WT: Wilms tumor; ASK: anaplastic sarcoma of the kidney; RMS: rhabdomyosarcoma.

## Supplementary Materials

The following are available online at https://www.mdpi.com/2072-6694/13/5/997/s1: Table S1: Search terms; Table S2: Reclassified patients; Table S3: Characteristics and outcome of CPDN patients based on well-documented reports; Table S4: Most frequently reported presenting signs and symptoms in pediatric patients with CPDN or CN (data available for 44/113 CPDN and 89/167 CN); Table S5: Characteristics and outcome of CN patients based on well-documented reports; Table S6: Individual data of cWT reports.
