# Supplementary material for: Clinical and Molecular Characteristics and Outcome of Cystic Partially Differentiated Nephroblastoma and Cystic Nephroma: A Narrative Review of the Literature
Source: Cancers (Basel). 2021 Feb 27;13(5):997. doi: 10.3390/cancers13050997 (PMC7957568; doi:10.3390/cancers13050997)
Supplement: Supplementary file 1 [file cancers-13-00997-s001.pdf]

Table S1: Search terms.

CPDN

**EMBASE**	cpdn:ti,ab OR nephroblastoma*:ti,ab AND (cystic:ti,ab OR cyst:ti,ab OR cysts:ti,ab OR ‘partially differentiated’:ti,ab OR 'kidney cyst'/exp)
**Pubmed**	CPDN[tiab] OR (nephroblastoma*[tiab] AND (cyst[tiab] OR cysts[tiab] OR cystic[tiab] OR “partially differentiated”[tiab] OR "Cysts"[Mesh]))

CN

**EMBASE**	cystic nephroma*:ti,ab
**Pubmed**	cystic nephroma*[tiab]

cWT

**EMBASE**	cyst*:ti,ab AND (wilm*:ti,ab OR nephroblastom*:ti,ab OR 'nephroblastoma'/exp)
**Pubmed**	(Wilm*[tiab] OR nephroblastoma*[tiab] OR "Wilms Tumor"[Mesh]) AND cyst*[tiab]

CPDN: cystic partially differentiated nephroblastoma; CN: cystic nephroma; cWT: cystic Wilms tumor.

Table S2. Reclassified patients. CPDN: cystic partially differentiated nephroblastoma; CN: cystic nephroma

Reference	Original classification	Reclassification	Based on
Van den Hoek et al, 2009 [1]	Cystic Wilms tumor	CPDN	Histological description, consulting pathologist
Andrews Jr et al, 1983 [2]	CPDN	CN	Histological description: 'No primitive stromal, blastemal or glomeruloid structures'
Fowler et al [3], 1971	CPDN/CN	CN	Histological description, original classification

Table S3. Characteristics and outcome of CPDN patients based on well-documented reports.

Report	N	Age Median in months (range)	Sex			Re-evaluated SIOP stage						Treatment				*DICER1* status / other relevant genetic findings	Outcome	
			M	F	*NA*	I	II	III	IV	V	*NA*	Pre-operative treatment	Complete nephrectomy	Partial nephrectomy	Post-operative treatment		Relapse	Death
**Saula et al. 2012** [4]	12	NA (0-4)	7	5	-	-	-	3	-	-	9	10 CT	12	-	7 CT<br>3 CT+RT	NA	-	2
**Luithle et al. 2007** [5]	7	10.8 (NA)	6	1	-	7	-	-	-	-	-	-	6	1	1 CT	NA	-	-
**Doros et al. 2014** [6]	6	7 (4-14)	2	4	-	-	-	-	-	-	6	*NA*	*NA*	*NA*	*NA*	**6 negative**	NA	NA
**Blakely et al. 2003** [7]	21	12 (5-43.6)	16	5	-	18	1	1	-	1	-	1 CT	19	2	13 CT	NA	-	-
**Joshi et al. 1990** [8]	11	6 (1-34)	4	7	-	11	-	-	-	-	-	-	10	1	5 CT<br>1 CT+RT	NA	-	-
**Joshi et al. 1989** [9]	18	12 (4-36)	12	4	2	14	-	1	-	1	2	-	17	1	6 CT	NA	1	1
**Kurian et al. 2018** [10]	4	27 (9-42)	3	1	-	2	-	1	-	1	-	2 CT	3	1	1 CT	NA	2	2
**Stout et al. 2016** [11]	1	18	1	-	-	-	-	1	-	1	-	1 CT	-	1	1 CT+RT	**1 negative**	-	-
**Odubanjo et al. 2014** [12]	1	30	1	-	-	-	-	1	-	-	-	1 CT	1	-	1 CT+RT	NA	-	-
**Mittal et al. 2013** [13]	1	2	-	1	-	-	-	-	-	-	1	-	1	-	*NA*	NA	-	-
**Ravi Kumar et al. 2010** [14]	1	17	1	-	-	-	-	-	-	-	1	-	1	-	*NA*	NA	-	-
**Van den Hoek et al. 2009** [1]	2	6.5 (5-8)	2	-	-	2	-	-	-	-	-	-	2	-	1 CT	NA	-	-
**Taskinen et al. 2009** [15]	1	7	-	1	-	1	-	-	-	-	-	-	-	1	1 CT	**Mulibrey Nanism** (Germline *TRIM37* mutation)	-	-
**Puvaneswary et al. 2006** [16]	1	30	1	-	-	-	-	-	-	-	1	*NA*	1	-	*NA*	NA	-	-
**Nayak et al. 2006** [17]	1	7.5	1	-	-	-	-	-	-	-	1	*NA*	1	-	-	NA	-	-
**Baker et al. 2008** [18]	1	18	1	-	-	-	-	1	-	-	-	-	1	-	*NA*	NA	1	-
**Singh et al. 2006** [19]	1	54	1	-	-	-	-	-	-	-	1	-	1	-	-	NA	-	-
**Tiryaki et al. 2003** [20]	1	9	1	-	-	1	-	-	-	-	-	*NA*	1	-	-	NA	-	-
**Rajangam et al. 2000** [21]	1	7	1	-	-	-	-	-	-	-	1	*NA*	1	-	1 CT	NA	-	-
**De Chadarévian et al. 1996** [22]	1	9	1	-	-	-	-	-	-	-	1	*NA*	1	-	*NA*	-	-	-

**Dey et al. 1996** [23]	1	18	-	1	-	-	-	-	-	-	1	1 CT	1	-	*NA*	NA	NA	NA
**Babut et al. 1993** [24]	3	12 (9-54)	1	2	-	-	-	-	-	-	3	1 CT	3	-	2 CT	NA	-	-
**Patriarca et al. 1992** [25]	1	30	-	1	-	1	-	-	-	-	-	1 CT	1	-	1 CT	NA	-	-
**Thambi Dorai et al. 1992** [26]	1	3	-	1	-	-	-	-	-	1	-	-	1	-	1 CT	NA	-	-
**Domizio et al. 1991** [27]	2	23 (13-33)	1	1	-	-	-	-	-	-	2	-	2	-	-	NA	-	-
**Kaneko et al. 1991** [28]	2	10.5 (3-18)	2	-	-	2	-	-	-	-	-	*NA*	*NA*	*NA*	*NA*	-	-	-
**Abara et al. 1990** [29]	1	7.5	1	-	-	-	-	-	-	-	1	-	1	-	-	NA	-	-
**Timmons et al. 1989** [30]	1	3	1	-	-	1	-	-	-	-	-	-	1	-	-	-	-	-
**Ugarte et al. 1981** [31]	2	4.5 (2-7)	2	-	-	1	-	-	-	-	-	*NA*	*NA*	*NA*	1 CT+RT	NA	-	-
**Joshi et al. 1977** [32]	3	15 (4-18)	2	1	-	-	-	-	-	-	3	-	3	-	-	NA	-	-
**Brown et al. 1975** [33]	1	9	1	-	-	-	-	-	-	-	1	1 CT	1	-	-	NA	-	-
**Cordeiro et al. 2020** [34]	1	34	-	1	-	-	-	-	-	-	1	1 CT	1	-	-	NA	-	-
**Furukawa et al. 2003** [35]	1	2	-	1	-	-	-	-	-	-	1	-	-	-	-	No *DICER1* mutation<br>Multiple variegated aneuploidy (MVA) and premature centromere division	-	1
**TOTAL**	113	12 (3 weeks - 4.5 years)	73	38	2	62	1	9	0	5	37	20	94	9	47<br>40 CT<br>7 RT+CT	7/7 negative for *DICER1*	4	6*

CPDN: cystic partially differentiated nephroblastoma; SIOP: International Society of Pediatric Oncology; N: number of patients; NA: not available; CT: chemotherapy; RT: radiotherapy

Table S4: Most frequently reported presenting signs and symptoms in pediatric patients with CPDN or CN (data available for 44/113 CPDN and 89/167 CN)

**Symptom**	**Frequency (percentage)**	
	**CPDN**	**CN**
Palpable/visible abdominal mass	40 (91%)	62 (70%)
Anaemia	8 (18%)*	0 (0%)
Hypertension	2 (4.5%)	4 (4.5%)
Asymptomatic / detected during screening	1 (2%)	7 (8%)
Haematuria	4 (9%)	6 (6.5%)
Abdominal/flank pain	3 (7%)	7 (8%)
Decreased appetite	1 (2%)	4 (4.5%)
Intussusception / constipation	0 (0%)	4 (4.5%)
Vomiting	0 (0%)	4 (4.5%)
Fever	1 (2%)	2 (2%)
Tachypnea	0 (0%)	1 (1%)
Weight loss	10 (23%)*	1 (1%)
Detoriation general condition	0 (0%)	1 (1%)
Renal insufficiency	0 (0%)	1 (1%)
**Total (cases)**	44	88

* Only mentioned in report describing CPDN in developing countries.

CPDN: cystic partially differentiated nephroblastoma; CN: cystic nephroma.

Table S5: Characteristics and outcome of CN patients based on well-documented reports

Report	N	Age (Median) in months (range)	Sex			Re-evaluated SIOP stage						Treatment				*DICER1* status / other relevant genetic findings	Concomitant tumors	Outcome	
			M	F	*NA*	I	II	III	IV	V	*NA*	Pre-operative treatment	Complete nephrectomy	Partial nephrectomy	Post-operative treatment			Relapse	Death
**Luithle et al, 2007** [5]	7	12.9	3	4	0	6	-	1	-	-	-	2 CT	6	1	-	NA	-	-	-
**Sarin et al, 2005** [36]	1	13	1	0	0	-	-	-	-	-	1	-	1	-	-	NA	-	-	-
**Kurian et al, 2018** [10]	1	4	1	0	0	-	-	-	-	1	-	-	-	1	-	NA	ERMS	-	-
**Van den Hoek et al, 2009** [1]	4	31	2	2	0	1	-	-	-	-	3	2 CT	3	1	-	NA	-	-	-
**Saskin et al, 2017** [37]	1	14	0	1	0	-	-	-	-	-	1	-	1	-	NA	Germline *DICER1* mutation	NBL<br>Nodular hyperplasia of thyroid	-	-
**Fernández-Martínez et al, 2017** [38]	1	11	0	1	0	-	-	-	-	-	1	-	1	-	NA	Germline *DICER1* mutation	PPB	-	-
**Tanaka et al, 2014** [39]	2	9	2	0	0	-	-	-	-	-	1	-	-	2	-	NA	NA	NA	NA
**Wani et al, 2012** [40]	1	11	1	0	0	-	-	-	-	-	1	-	1	-	NA	NA	NA	-	-
**Bhardwaj et al, 2011** [41]	1	30	1	0	0	-	-	-	-	1	-	-	NA	NA	NA	NA	PPB	NA	NA
**Shaheen et al, 2010** [42]	1	9	1	0	0	-	-	-	-	1	-	-	1	-	NA	NA	PPB<br>Duodenal polyp	-	-
**Taskinen et al, 2009** [15]	1	18	0	1	0	-	-	-	-	-	1	NA	NA	NA	NA	NA	PPB	-	-
**Agarwal et al, 2008** [43]	1	20	0	1	0	-	-	-	-	-	1	-	-	-	-	NA	PPB	-	1
**Deeg et al, 2008** [44]	1	102	1	0	0	-	-	-	-	-	1	-	1	-	-	NA	NA	NA	NA
**Boybeyi et al, 2008** [45]	4	15	2	2	0	-	-	-	-	-	4	1 CT	4	-	NA	NA	NA	-	-
**Silver et al, 2008** [46]	1	16	0	1	0	-	-	-	-	-	1	-	1	-	NA	NA	NA	NA	NA
**Somjee et al, 2003** [47]	1	16	0	1	0	-	-	-	-	-	1	-	1	-	NA	NA	-	-	-
**Lenz et al, 2005** [48]	1	16	1	0	0	-	-	-	-	1	-	NA	-	1	1 CT	NA	-	-	-
**Abt et al, 1979** [49]	1	18	0	1	0	-	-	-	-	-	1	-	1	-	NA	NA	Lung cyst	-	-
**Delahunt et al, 1993** [50]	2	29	1	1	0	-	-	-	-	-	2	-	2	-	NA	NA	-	-	-
**Mancini et al, 1986** [51]	1	20	0	1	0	-	-	-	-	-	1	-	1	-	1 CT	NA	-	-	-
**Ashley et al, 2007** [52]	2	36	2	0	0	-	-	-	-	1	1	-	1	1	-	NA	-	-	-
**Agnihotri et al, 2009** [53]	1	30	1	0	0	-	-	-	-	-	1	-	1	-	-	NA	NA	NA	NA
**Boggs 1956** [54]	2	8	2	0	0	-	-	-	-	-	1	-	2	-	-	NA	NA	-	1
**Sacher et al, 1997** [55]	2	18	2	0	0	-	-	-	-	-	2	NA	-	2	-	NA	NA	NA	NA
**De Kock et al, 2015** [56]	1	144	0	1	0	-	-	-	-	-	1	NA	-	1	-	Germline *DICER1* mutation	ERMS<br>Nodules with liver hyperplasia	NA	NA

																	Nodular hyperplasia of thyroid		
**Bouhafs et al, 2006** [57]	1	78	1	0	0	-	-	-	-	-	1	NA	1	-	NA	NA	-	-	-
**Gupta et al, 2007** [58]	1	7	0	1	0	-	-	-	-	-	1	NA	1	-	NA	NA	-	-	-
**Bal et al, 2005** [59]	2	34	1	1	0	-	-	-	-	-	1	NA	2	-	NA	NA	1 PPB	-	1
**Babut et al, 1993** [24]	4	16	2	2	0	-	-	-	-	-	4	2 CT+RT	3	1	-	NA	NA	NA	NA
**Cozzi et al, 2001** [60]	1	21	0	0	1	-	-	-	-	-	1	NA	-	1	-	NA	-	-	-
**De Kock et al, 2018** [61]	1	12	1	0	0	-	-	-	-	-	1	NA	1	-	NA	Germline *DICER1* mutation	Malignant teratoid CBME	-	1
**Cozzi et al, 2003** [62]	1	20	1	0	0	-	-	-	-	-	1	-	-	1	-	NA	-	-	-
**Nisen et al, 1987** [63]	1	30	1	0	0	-	-	-	-	-	1	-	1	-	NA	NA	NBL	-	-
**Yüksel et al, 2006** [64]	1	15	0	1	0	-	-	-	-	-	1	NA	1	-	NA	NA	Pulmonary introlobar sequestration	-	-
**Bardón-Cancho et al, 2017** [65]	1	30	0	1	0	-	-	-	-	-	1	-	-	1	NA	Somatic *DICER1* mutation	-	-	-
**Ishida et al, 2000** [66]	1	22	1	0	0	-	-	-	-	-	1	1 CT	-	-	NA	NA	PPB	-	-
**Gursev 2009** [67]	1	9	1	0	0	-	-	-	-	-	1	-	1	-	NA	NA	NA	NA	NA
**Aparanji et al, 2014** [68]	1	11	1	0	0	-	-	-	-	1	-	NA	-	1	NA	NA	NA	NA	NA
**Bouron-Dal Soglio et al, 2006** [69]	1	32	1	0	0	-	-	-	-	-	1	-	1	-	1 CT	NA	PPB	-	-
**Garrett et al, 1987** [70]	3	14	3	0	0	-	-	-	-	-	3	-	3	-	1 CT	NA	-	-	-
**Joshi et al, 1989** [9]	5	18	4	1	0	-	-	-	-	-	5	-	5	-	-	NA	NA	-	-
**Lanning et al, 1987** [71]	2	12.5	2	0	0	-	-	-	-	-	2	-	2	-	-	NA	NA	NA	NA
**Boulanger et al, 2003** [72]	1	18	0	1	0	-	-	-	-	-	1	-	1	-	NA	NA	-	-	-
**Hopkins et al, 2004** [73]	1	16	1	0	0	-	-	-	-	-	1	-	1	-	NA	NA	NA	NA	NA
**Makepeace et al, 2006** [74]	1	6	0	1	0	-	-	-	-	-	1	-	1	-	-	NA	NA	NA	NA
**Cajaiba et al, 2016** [75]	44	16	22	22	0	44	-	-	-	-	-	NA	41	3	NA	15 Somatic *DICER1* mutation	4 Pulmonary cysts	-	-
**Okada et al, 2003** [76]	1	90	0	1	0	-	-	-	-	-	1	-	-	1	NA	NA	-	-	-
**Jenkner et al, 2001** [77]	3	31	3	0	0	-	-	-	-	-	3	NA	3	-	-	NA	-	-	-
**Ferrer et al, 1994** [78]	1	54	1	0	0	-	-	-	-	1	-	-	-	1	-	NA	-	-	-
**Drut et al, 1992** [79]	1	42	1	0	0	-	-	-	-	-	1	-	1	-	-	NA	NA	NA	NA
**Masieri et al, 2019** [80]	1	114	0	0	1	-	-	-	-	-	1	-	-	1	-	NA	-	-	-
**Hammon et al, 2003** [81]	1	30	1	0	0	-	-	-	-	-	1	-	1	-	-	NA	-	-	-
**Effert et al, 1999** [82]	1	Prenatal	1	0	0	-	-	-	-	-	1	NA	1	-	NA	NA	NA	NA	NA
**Huang et al, 2016** [83]	1	8	0	1	0	-	-	-	-	-	1	-	1	-	-	NA	NA	NA	NA
**Singh et al, 2006** [84]	1	6	0	1	0	-	-	-	-	-	1	-	1	-	-	NA	NA	NA	NA
**Faure et al, 2016** [85]	2	60	1	1	0	-	-	-	-	-	2	-	2	-	-	2 Germline *DICER1* mutation	1 Sertolli-leydig cell tumor	NA	NA
**Karmazyn et al, 2015** [86]	1	86	1	0	0	-	-	-	-	-	1	-	1	-	-	NA	NA	NA	NA

**Li et al, 2017** [87]	7	16	5	1	0	-	-	-	-	1	6	-	NA	NA	-	6 Somatic *DICER1* mutation	NA	NA	NA
**Billiet et al, 1988** [88]	2	14	1	1	0	-	-	-	-	-	-	-	-	2	-	NA	NA	1	-
**Gallo et al, 1977** [89]	4	20	1	3	0	-	-	-	-	-	4	1 RT	4	-	-	NA	1 WT	-	1
**Chan et al, 1996** [90]	1	15	1	0	0	-	-	-	-	-	1	-	1	-	-	NA	-	-	-
**Thijssen et al, 1989** [91]	2	16	1	1	0	-	-	-	-	-	1	-	2	-	NA	NA	NA	NA	NA
**Domizio et al, 1991** [27]	1	24	1	0	0	-	-	-	-	-	1	-	1	-	-	NA	-	-	-
**Staicu et al, 2016** [92]	1	Prenatal	0	1	0	-	-	-	-	-	1	-	-	-	-	NA	-	-	1
**Kousari et al, 2014** [93]	1	5	0	1	0	-	-	-	-	-	1	-	-	1	NA	NA	PPB	-	-
**Boman et al, 2006** [94]	3	11	0	3	0	-	-	-	-	1	2	NA	NA	NA	NA	NA	3 PPB<br>1 Small bowel polyp	-	-
**Bueno et al, 2017** [95]	2	45.5	1	1	0	-	-	-	-	-	2	NA	NA	NA	NA	2 Germline *DICER1* mutation	1 Pineal cyst<br>1 ERMS, fibroadenomas, thyroid cysts and exostoses	NA	NA
**Patnayak et al, 2008** [96]	1	198	0	1	0	-	-	-	-	-	1	NA	1	-	NA	NA	NA	NA	NA
**Vujaniç et al, 2000** [97]	1	21	0	1	0	-	-	-	-	-	1	1 CT	1	-	NA	NA	WT	NA	NA
**Andrews et al, 1983** [2]	1	6.5	1	0	0	-	-	-	-	-	1	-	1	-	-	NA	-	-	-
**Wu et al, 2016** [98]	1	7	0	1	0	-	-	-	-	-	1	-	NA	NA	NA	Germline *DICER1* mutation	ASK	-	-
**Frazier et al, 1951** [99]	2	24	2	0	0	-	-	-	-	-	1	1 RT	2	-	-	NA	-	-	-
**Dainko et al, 1963** [100]	1	22	0	1	0	-	-	-	-	-	1	-	1	-	NA	NA	-	-	-
**Fowler et al, 1971** [3]	1	13	1	0	0	-	-	-	-	-	1	1 RT	1	-	NA	NA	-	-	-
**Apellaniz-Ruiz et al, 2020** [101]	1	10	1	0	0	-	-	-	-	-	1	-	-	1	NA	Germline *DICER1* mutation	Paratesticular sarcoma	-	-
**Dural et al, 2020** [102]	1	12	0	1	0	-	-	-	-	-	1	-	1	-	NA	Germline *DICER1* mutation	ERMS	-	-
**Loomis et al, 2020** [103]	1	22	0	1	0	-	-	-	-	-	1	-	1	-	-	NA	NA	NA	NA
**Faria et al, 1996** [104]	1	26	0	1	0	-	-	-	-	-	1	-	1	-	1 CT	NA	Anaplastic malignant mesenchymoma	-	-
**TOTAL**	167	16	94	72	2	51	-	1	-	9	107	12	121	27	5 CT	12 germline<br>27 somatic	26 patients	1	6

CN: cystic nephroma; N: number of patients; SIOP: International Society of Pediatric Oncology; NA: not available; CT: chemotherapy; RT: radiotherapy; ERMS: embryonal rhabdomyosarcoma; NBL: neuroblastoma; PPB: pleuropulmonary blastoma; WT: Wilms tumor; ASK: anaplastic sarcoma of the kidney

Table S6: Individual data of cWT reports

Report	N	Age (Median) in months (range)	Sex			Re-evaluated SIOP stage						Treatment				*DICER1* status / other relevant genetic findings	Concomitant tumors	Outcome	
			M	F	*NA*	I	II	III	IV	V	*NA*	Pre-operative treatment	Complete nephrectomy	Partial nephrectomy	Post-operative treatment			Relapse	Death
**Kurose et al, 2018** [105]	**1**	6	0	1	0	-	-	1	-	-	-	-	1	-	1 CT+RT	NA	-	-	-
**Kurian et al, 2018** [10]	**4**	10	4	0	0	4	-	-	-	-	-	-	3	1	3 CT	NA	-	-	-
**Bindhu et al, 2010** [106]	**1**	9.5	0	0	1	-	-	-	1	-	-	1 CT	1	-	1 CT	NA	-	-	-
**Babut et al, 1993** [24]	**1**	42	1	0	0	-	-	-	1	-	-	1 CT	1	-	1 CT	NA	-	-	-
**Domizio et al, 1991** [27]	**1**	10	1	0	0	-	-	-	-	-	1	-	-	1	-	Denys Drash syndrome	-	-	-
**Jenkins et al, 1991** [107]	**2**	10	1	1	0	-	-	-	-	-	2	-	2	-	1 CT	NA	-	-	-
**Reinberg et al, 1988** [108]	1	5	0	1	0	1	-	-	-	-	-	NA	-	1	1 CT	VATER-association	-	-	-
**Andrews et al, 1983** [2]	1	4	1	0	0	-	-	-	-	-	1	-	1	-	1 CT	NA	-	-	-
**Wood et al, 1982** [109]	1	15	1	0	0	1	-	-	-	-	-	-	1	-	1 CT	NA	-	-	-
**Datnow et al, 1976** [110]	1	7	1	0	0	-	-	-	-	-	1	-	1	-	1 CT	-	-	-	-
**Ariel et al, 1996** [111]	1	30	0	1	0	-	-	-	-	-	1	-	2	-	NA	NA	NA	NA	NA
**Nakamura et al, 1981** [112]	1	9	0	1	0	-	-	-	-	1	-	-	-	-	-	NA	Vaginal botryoid sarcoma	-	1
**Keegan et al, 1979** [113]	1	2	1	0	0	-	-	-	-	-	1	-	1	-	1 CT	NA	-	-	-
**Nakamura et al, 1985** [114]	1	16	0	1	0	-	-	-	-	1	-	-	-	-	-	Karyotype 46,XX/47,XX+8 mosaicism	-	-	1
**TOTAL**	1 8	10	11	6	1	6	-	1	2	2	7	2 CT	13	3	11 CT 1 CT+RT	In 3 patients	In 1 patient	-	2

cWT: cystic Wilms tumor; N: number of patients; SIOP: International Society of Pediatric Oncology; CT: chemotherapy; RT: radiotherapy; NA: not available

References

1. Van Den Hoek, J.; De Krijger, R.; Van De Ven, K.; Lequin, M.; Van Den Heuvel-Eibrink, M.M. Cystic nephroma, cystic partially differentiated nephroblastoma and cystic Wilms' tumor in children: A spectrum with therapeutic dilemmas. *Urol. Int.* **2009**, *82*, 65–70.
2. Andrews Jr, M.J.; Askin, F.B.; Fried, F.A.; McMillan, C.W.; Mandell, J. Cystic partially differentiated nephroblastoma and polycystic Wilms tumor: A spectrum of related clinical and pathologic entities. *J Urol.* **1983**, *129*, 577–580.
3. Fowler, M. Differentiated nephroblastoma: Solid, cystic or mixed. *J Pathol.* **1971**, *105*, 215–218.
4. Saula, P.W.; Hadley, G.P. Pediatric non-wilms' renal tumors: A third world experience. *World J. Surg.* **2012**, *36*, 565–572.
5. Luithle, T.; Szavay, P.; Furtwängler, R.; Graf, N.; Fuchs, J. Treatment of Cystic Nephroma and Cystic Partially Differentiated Nephroblastoma-A Report From the SIOP/GPOH Study Group. *J. Urol.* **2007**, *177*, 294–296.
6. Doros, L.A.; Rossi, C.T.; Yang, J.; Field, A.; Williams, G.M.; Messinger, Y.; Cajaiba, M.M.; Perlman, E.J.; Schultz, K.A.; Cathro, H.P.; et al. DICER1 mutations in childhood cystic nephroma and its relationship to DICER1-renal sarcoma. *Mod. Pathol.* **2014**, *27*, 1267–1280.
7. Blakely, M.L.; Shamberger, R.C.; Norkool, P.; Beckwith, J.B.; Green, D.M.; Ritchey, M.L. Outcome of children with cystic partially differentiated nephroblastoma treated with or without chemotherapy. *J. Pediatr. Surg.* **2003**, *38*, 897–900.
8. Joshi, V.V.; Beckwith, J.B. Pathologic delineation of the papillonodular type of cystic partially differentiated nephroblastoma. A review of 11 cases. *Cancer* **1990**, *66*, 1568–1577.
9. Joshi, V.V.; Beckwith, J.B. Multilocular cyst of the kidney (cystic nephroma) and cystic, partially differentiated nephroblastoma. Terminology and criteria for diagnosis. *Cancer* **1989**, *64*, 466–479.
10. Kurian, J.J.; Jehangir, S.; Korula, A. Multiloculated Cystic Renal Tumors of Childhood: Has the Final Word Been Spoken. *J. Indian Assoc. Pediatr. Surg.* **2018**, *23*, 22–26.
11. Stout, T.E.; Au, J.K.; Hicks, J.M.; Gargollo, P.C. A Case of Bilateral Cystic Partially Differentiated Nephroblastoma vs Cystic Wilms' Tumor: Highlighting a Diagnostic Dilemma. *Urology* **2016**, *92*, 106–109.
12. Odubanjo, M.O.; Ademuyiwa, A.O.; Daramola, A.O.; Orah, N.O.; Elebute, O.A.; Abdulkareem, F.B.; Akinda, O.R.. Cystic poorly differentiated nephroblastoma: A case report and review of literature. *African J. Urol.* **2014**, *20*, 144–148.
13. Mittal, M.K.; Sureka, B.; Sinha, M.; Thukral, B.B. Cystic partially differentiated nephroblastoma: A rare renal tumor. *Indian J Nephrol.* **2013**, *23*, 460–461.
14. Ravi Kumar, V.R.; Rajamani, G.; Raman, M.L. Cystic partially differentiated nephroblastoma with ureteric extension. *Indian J. Pathol. Microbiol.* **2010**, *53*, 831–832.
15. Taskinen, S.; Lohi, J.; Kivisaari, R.; Fagerholm, R.; Rintala, R.; Taskinen, M. Segmental cystic kidney tumours in children. *Scand. J. Urol. Nephrol.* **2009**, *43*, 476–481.
16. Puvaneswary, M.; Macintosh, J.; Cassey, J. Cystic partially differentiated nephroblastoma. *Australas. Radiol.* **2006**, *50*, 255–257.
17. Nayak, A.; Iyer, V.K.; Agarwala, S.; Verma, K. Fine needle aspiration cytology of cystic partially differentiated nephroblastoma of the kidney. *Cytopathology* **2006**, *17*, 145–148.
18. Baker, J.M.; Viero, S.; Kim, P.C.; Grant, R.M. Stage III cystic partially differentiated nephroblastoma recurring after nephrectomy and chemotherapy. *Pediatr. Blood Cancer* **2008**, *50*, 129–131.
19. Singh, S.; Gupta, R.; Khurana, N. Cystic partially differentiated nephroblastoma: A rare differentiated variant of Wilm's tumour. *J. Postgrad. Med.* **2006**, *52*, 45–46.
20. Tiryaki, T.; Hücümenoğlu, S.; Livanelioğlu, Z.; Atayurt, H. Cystic partially differentiated nephroblastoma: A case report. *Urol. Int.* **2003**, *70*, 223–226.
21. Rajangam, K.; Narasimhan, K.L.; Trehan, A.; Rawal, A.; Radotra, B.; Rao, K.L. Partial nephrectomy in cystic partially differentiated nephroblastoma. *J. Pediatr. Surg.* **2000**, *35*, 510–512.
22. De Chadarévian, J.P.; Punnett, H.H.; Billmire, D.F.; Tomczak, E.Z. Hyperdiploidy and trisomy 12 in the cystic partially differentiated nephroblastoma. *Hum. Pathol.* **1996**, *27*, 980–981.
23. Dey, P.; Das, A.; Radhika, S. Fine needle aspiration cytology of cystic partially differentiated nephroblastoma: A case report. *Acta Cytol.* **1996**, *40*, 770–772.
24. Babut, J.M.; Bawab, F.; Jouan, H.; Coeurdacier, P.; Treguier, C.; Fremond, B. Renal cystic tumours in children—A diagnostic challenge. *Eur. J. Pediatr. Surg.* **1993**, *3*, 157–160.
25. Patriarca, C.; Orazi, A.; Massimino, M.; Luksch, R. A cystic partially differentiated nephroblastoma producing α-fetoprotein. *Am. J. Pediatr. Hematol. Oncol.* **1992**, *14*, 352–355.
26. Thambi Dorai, C.R.; Boucaut, H.A.P.; Le Quesne, G.W.; Toogood, I.J.R.; Bourne, A.J.; Byard, R.W. Unilateral cystic, partially-differentiated nephroblastoma with bilateral nephroblastomatosis. *Pediatr. Surg. Int.* **1994**, *9*, 137–140.

27. Domizio, P.; Risdon, R.A. Cystic renal neoplasms of infancy and childhood: A light microscopical, lectin histochemical and immunohistochemical study. *Histopathology* **1991**, *19*, 199–209.
28. Kaneko, Y.; Homma, C.; Maseki, N.; Sakurai, M.; Hata, J. Correlation of chromosome abnormalities with histological and clinical features in Wilms' and other childhood renal tumors. *Cancer Res.* **1991**, *51*, 5937–5942.
29. Abara, O.E.; Liu, P.; Churchill, B.M.; Mancer, K. Magnetic resonance imaging of cystic,partially differentiated nephroblastoma. *Urology* **1990**, *36*, 424–427.
30. Timmons, C.F.; McGavran, L.; Unterkircher, L.; Beckwith, J.B.; Wilson, H.L. Hyperdiploidy including trisomy 8 in a cystic partially differentiated nephroblastoma. *Cancer Genet. Cytogenet.* **1989**, *41*, 79–85.
31. Ugarte N, Gonzalez-Crussi F, Hsueh, W. Wilms' tumor: Its morphology in patients under one year of age. *Cancer* **1981**, *48*, 346–353.
32. Joshi, V.V.; Banerjee, A.K.; Yadav, K.; Pathak, I.C. Cystic partially differentiated nephroblastoma. A clinicopathologic entity in the spectrum of infantile renal neoplasia. *Cancer* **1977**, *40*, 789–795.
33. Brown, J.M. Cystic partially differentiated nephroblastoma. *J. Pathol.* **1975**, *115*, 175–178.
34. Cordeiro, L.P.V.; Carvalho, A.C.M.; Silva, I.M.; Martins, F.P.; Amaro, A.P.; Carvalho, E.M. Cystic partially differentiated nephroblastoma: A rare pediatric renal tumor—Case report. *Radiol. Case Rep.* **2020**, *15*, 1133–1137.
35. Furukawa, T.; Azakami, S.; Kurosawa, H.; Ono, Y.; Ueda, Y.; Konno, Y. Cystic Partially Differentiated Nephroblastoma, Embryonal Rhabdomyosarcoma, and Multiple Congenital Anomalies Associated with Variegated Mosaic Aneuploidy and Premature Centromere Division: A Case Report. *J. Pediatr. Hematol. Oncol*. **2003**, *25*, 896–899.
36. Sarin, Y.K.; Sengar, M. Cystic nephroma. *Indian Pediatr.* **2005**, *42*, 84–86.
37. Saskin, A.; de Kock, L.; Sabbaghian, N.; Apellaniz-Ruiz, M.; Bozkurt, C.; Bouron-Dal Soglio, D.; Foulkes, W.D. A case of neuroblastoma in DICER1 syndrome: Chance finding or noncanonical causation? *Pediatr. Blood Cancer* **2018**, *65*, e26715.
38. Fernández-Martinez, L.; Villegas, J.A.; Santamaria, I.; Pitiot, A.S.; Alvarado, M.G.; Fernandez, S.; Torres, H.; Paredes, A.; Blay, P.; Balbín, M. Identification of somatic and germ-line DICER1 mutations in pleuropulmonary blastoma, cystic nephroma and rhabdomyosarcoma tumors within a DICER1 syndrome pedigree. *BMC Cancer* **2017**, *17*, 146.
39. Tanaka, Y.; Uchida, H.; Kawashima, H.; Masuko, T.; Takazawa, S.; Deie, K. Laparoscopic partial nephrectomy for the treatment of large cystic nephroma in children. *J. Laparoendosc. Adv. Surg. Tech.* **2014**, *24*, 901–906.
40. Wani, B.; Kolte, G.; Rathod, V. Asymptomatic Infantile Cystic Nephroma: A Diagnostic Dilemma. *Indian J. Surg.* **2012**, *74*, 501–503.
41. Bhardwaj, A.K.; Sharma, P.D.; Mittal, A.; Sharma, A. Bilateral cystic nephroma with pleuropulmonary blastoma. *BMJ Case Rep.* **2011**, *2011*, bcr0520114171.
42. Shaheen, I.S.; Fitzpatrick, M.; Brownlee, K.; Bhuskute, N.; Elliott, M.; Powis, M.; Ahmad, N.; Tyerman, K. Bilateral progressive cystic nephroma in a 9-month-old male infant requiring renal replacement therapy. *Pediatr. Nephrol.* **2010**, *25*, 1755–1758.
43. Agarwal, A.K.; Das, S.; Agarwal, A.; Ghosh, D.; Chaterjee, N.; Pal, M.S. Pleuropulmonary blastema with cystic nephroma—A rare presentation and surgical dilemma. *Indian J. Pediatr.* **2008**, *75*, 1266–1268.
44. Deeg, K.H.; Gerdemann, C.; Weingärtner, K.; Seitz, G. Sonographic diagnosis of an unusual case of multilocular cystic nephroma mimicking polycystic kidney disease. *Ultraschall. Med.* **2008**, *29* (Suppl. 5), 264–267.
45. Boybeyi, O.; Karnak, I.; Orhan, D.; Ciftci, A.O.; Tanyel, F.C.; Kale, G.; Şenocak, M.E. Cystic nephroma and localized renal cystic disease in children: Diagnostic clues and management. *J. Pediatr. Surg.* **2008**, *43*, 1985–1989.
46. Silver, I.M.F.; Boag, A.H.; Soboleski, D.A. Best cases from the AFIP: Multilocular cystic renal tumor: cystic nephroma. *Radiographics.* **2008**, *28*, 1221-1225.
47. Somjee, S.; Jindel, R.; Advani, S.H. Benign multilocular cystic nephroma. *Indian J. Pediatr.* **2003**, *70*, 515–517.
48. Lenz, M.P.; Warmann, S.W.; Scheel-Walter, H.G.; Schafer, J.; Wehrmann, M.; Hacker, H.W.; Fuchs, F. A complicated case of bilateral cystic nephroma in a 16-month-old boy. *Pediatr. Surg. Int.* **2005**, *21*, 1011–1014.
49. Abt, A.B.; Demers, L.M.; Shochat, S.J. Cystic nephroma: An ultrastructural and biochemical study. *J. Urol.* **1979**, *122*, 539–541.
50. Delahunt, B.; Thomson, K.J.; Ferguson, A.F.; Neale, T.J.; Meffan, P.J.; Nacey, J.N. Familial cystic nephroma and pleuropulmonary blastoma. *Cancer* **1993**, *71*, 1338–1342.
51. Mancini, A.F.; Zanetti, G.; Rosito, P.; Capucci, M.C.; Federici, S.; Malossi, R.; Di Caro, A. Cystic nephroma in children. Report of a case. *Tumori* **1986**, *72*, 99–104.
52. Ashley, R.A.; Reinberg, Y.E. Familial Multilocular Cystic Nephroma: A Variant of a Unique Renal Neoplasm. *Urology* **2007**, *70*, e9–e179.
53. Agnihotri, S.; Jeebun, N.; Ramputty, P. Multicystic nephroma: A rare entity. *J. Nephrol.* **2009**, *22*, 411–413.

54. Boggs, L.K.; Kimmelstiel, P. Benign multilocular cystic nephroma: Report of two cases of so-called multilocular cyst of the kidney. *J. Urol.* **1956**, *76*, 530–541.
55. Sacher, P., Willi, U.V.; Niggli, F. Cystic nephroma: A rare benign renal tumor. *Pediatr. Surg. Int.* **1998**, *13*, 197–199.
56. de Kock, L.; Druker, H.; Weber, E.; Hamel, N.; Traubici, J.; Malkin, D.; Arseneau, J.; Stewart, C.J.R.; Bouron-Dal Soglio, D.; Priest, J.R.; et al. Ovarian embryonal rhabdomyosarcoma is a rare manifestation of the DICER1 syndrome. *Hum. Pathol.* **2015**, *46*, 917–922.
57. Bouhafs, A.; Cherradi, N.; Lamaalmi, N.; Belkacem, R.; Barahioui, M. An unusual case of multilocular cystic nephroma with prominent renal pelvis involvement. *Case Rep. Int. J. Urol.* **2006**, *13*, 436–438.
58. Gupta, R.; Dhingra, K.; Singh, S.; Nigam, S.; Jain, S. Multicystic nephroma: A case report. *Acta Cytol.* **2007**, *51*, 651–653.
59. Bal, N.; Kayaselcuk, F.; Polat, A.; Bolat, F.; Yilmaz, Z.; Tuncer, I. Familial cystic nephroma in two siblings with pleuropulmonary blastoma. *Pathol. Oncol. Res.* **2005**, *11*, 53–56.
60. Cozzi, D.A.; Schiavetti, A.; Morini, F.; Castello, M.A.; Cozzi, F. Nephron-sparing surgery for unilateral primary renal tumor in children. *J Pediatr. Surg.* **2001**, *36*, 362–365.
61. de Kock, L.; Geoffrion, D.; Rivera, B.; Wagener, R.; Sabbaghian, N.; Bens, S.; Ellezam, B.; Bouron-Dal Soglio, D.; Ordóñez, J.; Sacharow, S.; et al. Multiple DICER1-related tumors in a child with a large interstitial 14q32 deletion. *Genes Chromosomes Cancer* **2018**, *57*, 223–230.
62. Cozzi, F.; Morini, F.; Schiavetti, A.; Catalano, C.; Bosco, S.; Cozzi, D.A. Enucleative surgery in an infant with giant cystic nephroma. *J. Urol.* **2003**, *169*, 1493–1494.
63. Nisen, P.D.; Rich, M.A.; Gloster, E.; Valderrama, E.; Saric, O.; Shende, A.; Lanzkowsky, P.; Alt, F.W. N-myc oncogene expression in histopathologically unrelated bilateral pediatric renal tumors. *Cancer* **1988**, *61*, 1821–1826.
64. Yüksel, S.; Ekim, M.; Fitöz, S.; Soygür, T.; Sertçelik, A.; Perçinel, S.; Acar, B.; Özçakar, Z.B.; Comba, A.; Yalçınkaya, F. The association of cystic nephroma with pulmonary sequestration: Is it a coincidence or not? *Pediatr. Nephrol.* **2006**, *21*, 1041–1044.
65. Bardón-Cancho, E.J.; Haro-Diaz, A.; Alonso-Garcia-de la Rosa, F.J.; Huerta-Aragones, J.; Garcia-Morin, M.; Gonzalez-Martinez, F.; Garrido-Colino, C. DICER1 mutation and tumors associated with a familial tumor predisposition syndrome: Practical considerations. *Fam. Cancer* **2017**, *16*, 291–294.
66. Ishida, Y.; Kato, K.; Kigasawa, H.; Ohama, Y.; Ijiri, R.; Tanaka, Y. Synchronous occurrence of pleuropulmonary blastoma and cystic nephroma: Possible genetic link in cystic lesions of the lung and the kidney. *Med. Pediatr. Oncol.* **2000**, *35*, 85–87.
67. Gursev; Sarda, D.; Joshi, P.; Ahmad, A.; Kothari, P. Cystic nephroma in childhood. *Afr. J. Paediatr. Surg*. **2009**, *6*, 69–70.
68. Aparanji, G.; Agarwal, I.; Chaturvedi, S. Quiz Page JULY 2014: Bilateral abdominal masses in an infant. *Am. J. Kidney Dis.* **2014**, *64*, xvii–xix.
69. Bouron-Dal Soglio, D.; Harvey, I.; Yazbeck, S.; Rypens, F.; Oligny, L.L.; Fournet, J.C. An association of pleuropulmonary blastoma and cystic nephroma: Possible genetic association. *Pediatr. Dev. Pathol.* **2006**, *9*, 61–64.
70. Garrett, A.; Carty, H.; Pilling, D. Multilocular cystic nephroma: Report of three cases. *Clin. Radiol.* **1987**, *38*, 55–57.
71. Lanning, P.; Lanning, M.; Heikkinen, E.; Kinnunen, P. Multilocular cystic nephroma simulating Wilms' tumour. *Rofo* **1987**, *147*, 463–465.
72. Boulanger, S.C.; Brisseau, G.F. Cystic nephroma: A benign renal tumor of children and adults. *Surgery* **2003**, *133*, 596–597.
73. Hopkins, J.K.; Giles, H.W.; Wyatt-Ashmead, J.; Bigler, S.A. Best cases from the AFIP. Cystic nephroma. *Radiographics* **2004**, *24*, 589–593.
74. Makepeace, J.C.; Sellaturay, S.; Mushtaq, I.; Sebire, N.J. Benign cystic nephroma. *Br. J. Hosp. Med.* **2006**, *67*, 45.
75. Cajaiba, M.M.; Khanna, G.; Smith, E.A.; Gellert, L.; Chi, Y.Y.; Mullen, E.A.; Hill, D.A.; Geller, J.I.; Dome, J.S.; Perlman, E.J. Pediatric cystic nephromas: Distinctive features and frequent DICER1 mutations. *Hum. Pathol.* **2016**, *48*, 81–87.
76. Okada, T.; Yoshida, H.; Matsunaga, T.; Kouchi, K.; Ohtsuka, Y.; Saitou, T.; Horie, H.; Ohnuma, n. Nephron-Sparing Surgery for Multilocular Cyst of the Kidney in a Child. *J. Pediatr. Surg*. **2003**, *38*, 1689–1692.
77. Jenkner, A.; Camassei, F.D.; Boldrini, R.; de Sio, L.; Ravà, L.; Bosman, C.; Boglino, C.; Donfrancesco, A. 111 Renal neoplasms of childhood: A clinicopathologic study. *J. Pediatr. Surg.* **2001**, *36*, 1522–1527.
78. Ferrer, F.A.; McKenna, P.H. Partial nephrectomy in a metachronous multilocular cyst of the kidney (cystic nephroma). *J. Urol*. **1994**, *151*, 1358–1360.
79. Drut, R. Cystic nephroma: Cytologic findings in fine-needle aspiration cytology. *Diagn. Cytopathol.* **1992**, *8*, 593–595.

80. Masieri, L.; Sessa, F.; Cini, C.; Sessa, M.; Vanacore, D.; Tasso, G.; Pili, A.; Sforza, S.; Greco, I.; Campi, R.; et al. Robot-assisted nephron-sparing surgery for cystic nephroma in a pediatric patient: A case report. *J. Endourol. Case Rep*. **2019**, *5*, 7–9.
81. Hammon, G.; Deeg, K.H.; Wolf, A.; Seitz, G.; Spindler-Thiele, S. Multilocular cystic nephroma. A rare benign renal tumor in childhood. *Monatsschr. Kinderheilkd*. **2003**, *151*, 308–310.
82. Effert, P.J.; Röttger, P.; Steffens, J. Cystic nephroma. *Urol. Ausgabe B* **1999**, *39*, 499–501.
83. Huang, S.H.; Chen, T.J. Cystic nephroma of childhood: A case report and review of the literature. *Urol. Sci.* **2016**, *27*, 171–173.
84. Singh, S.; Chowdhury, V.; Dixit, R.; Manchanda, A. Multilocular cystic nephroma of the kidney: A case report. *Indian J. Radiol. Imaging*. **2006**, *16*, 901–904.
85. Faure, A.; Atkinson, J.; Bouty, A.; O'Brien, M.; Levard, G.; Hutson, J.; Heloury, Y. DICER1 pleuropulmonary blastoma familial tumour predisposition syndrome: What the paediatric urologist needs to know. *J. Pediatr. Urol.* **2016**, *12*, 5–10.
86. Karmazyn, B.; Tawadros, A.; Delaney, L.R.; Marine, M.B.; Cain, M.P.; Rink, R.C.; Jennings, S.G.; Kaefer, M. Ultrasound classification of solitary renal cysts in children. *J. Pediatr. Urol*. **2015**, *11*, e1–e149.
87. Li, Y.; Pawel, B.R.; Hill, D.A.; Epstein, J.I.; Argani, P. Pediatric Cystic Nephroma Is Morphologically, Immunohistochemically, and Genetically Distinct From Adult Cystic Nephroma. *Am. J. Surg. Pathol*. **2017**, *41*, 472–481.
88. Billiet, I.; Van Poppel, H.; Baert, L. Actual approach to cystic nephroma. *Eur Urol.* **1988**, *14*, 280–286.
89. Gallo, G.E.; Penchansky, L. Cystic nephroma. *Cancer* **1977**, *39*, 1322–1327.
90. Chan, K.L.; Chan, K.W.; Peh, W.C. Clinics in diagnostic imaging (18). Multilocular cystic nephroma. *Singapore Med. J.* **1996**, *37*, 536–540.
91. Thijssen, A.M.; Carpenter, B.; Jiminez, C.; Schillinger, J. Multilocular cyst (multilocular cystic nephroma) of the kidney: A report of 2 cases with an unusual mode of presentation. *J. Urol.* **1989**, *142*, 346–348.
92. Staicu, A.; Popa-Stanila, R.; Gheban, D.; Chiriac, L.; Turcu, F.R.; Caracostea, G.; Stamatian, F. Imagistic and histopathological description of a cystic nephroma during early second trimester of gestation. Case report. *Med. Ultrason*. **2016**, *19*, 327–329.
93. Kousari, Y.M.; Khanna, G.; Hill, D.A.; Dehner, L.P. Case 211: Pleuropulmonary blastoma in association with cystic nephroma-DICER1 syndrome. *Radiology* **2014**, *273*, 622–625.
94. Boman, F.; Hill, D.A.; Williams, G.M.; Chauvenet, A.; Fournet, J.C.; Bouron-dal Soglio, D.; Messinger, Y.; Priest, J.R. Familial association of pleuropulmonary blastoma with cystic nephroma and other renal tumors: A report from the International Pleuropulmonary Blastoma Registry. *J. Pediatr*. **2006**, *149*, 850–854.
95. Bueno, M.T.; Martinez-Rios, C.; de la Puente Gregorio, A.; Ahyad, R.A.; Villani, A.; Druker, H.; van Engelen, K.; Gallinger, B.; Aronoff, L.; Grant, R.; et al. Pediatric imaging in DICER1 syndrome. *Pediatr Radiol.* **2017**, *47*, 1292–1301.
96. Patnayak, R.; Reddy, M.K.; Subramanian, S.; Ravisankar, G. Multilocular cystic nephroma of the kidney. *Indian J. Pathol. Microbiol.* **2008**, *51*, 563–565.
97. Vujaniç, G.M.; Jenney, M.E.; Adams, H.; Meyrick, S.M. Juxtaposed cystic nephroma and Wilms' tumor. *Pediatr. Dev. Pathol.* **2000**, *3*, 91–94.
98. Wu, M.K.; Cotter, M.B.; Pears, J.; McDermott, M.B.; Fabian, M.R.; Foulkes, W.D.; O'Sullivan, M.J. Tumor progression in *DICER1*-mutated cystic nephroma—witnessing the genesis of anaplastic sarcoma of the kidney. *Hum Pathol.* **2016**, 53, 114-120.
99. Frazier, T.H. Multilocular cysts of the kidney. *J. Urol.* **1951**, *65*, 351–363.
100. Dainko, E.A.; Dammers, W.R.; Economou, S.G. Multilocular cysts of the kidney in children: Report of a case and review of the literature. *J. Pediatr.* **1963**, *63*, 249–255.
101. Apellaniz-Ruiz, M.; Cullinan, N.; Grant, R.; Marrano, P.; Priest, J.R.; Thorner, P.S.; Goudie, C.; Foulkes, W.D. DICER1 screening in 15 paediatric paratesticular sarcomas unveils an unusual DICER1-associated sarcoma. *J. Pathol. Clin. Res.* **2020**, *6*, 185–194.
102. Dural, O.; Kebudi, R.; Yavuz, E.; Yilmaz, I.; Buyukkapu Bay, S.; Schultz, K.A.P.; Hill, D.A. DICER1-Related Embryonal Rhabdomyosarcoma of the Uterine Corpus in a Prepubertal Girl. *J. Pediatr. Adolesc. Gynecol*. **2020**, *33*, 173–176.
103. Loomis, J.; Peard, L.; Walker, J.; Cost, N.G.; Saltzman, A.F. Open Radical Nephrectomy for Suspected Renal Malignancy–Tips and Tricks. *Urology* **2019**, *133*, 241–242.
104. Faria, P.A.; Zerbini, M.C. Dedifferentiated cystic nephroma with malignant mesenchymoma as the dedifferentiated component. *Pediatr. Pathol. Lab. Med. J. Soc. Pediatr. Pathol. Affil. Int. Paediatr. Pathol. Assoc.* **1996**, *16*, 1003–1011.
105. Kurose, N.; Takenaka, M.; Yamashita, M.; Shimaguchi, C.; Nakano, M.; Britni, B.; Guo, X.; Futatsuya, C.; Shioya, A.; Yamada, S. A case report of infantile cystic nephroblastoma. *Diagn. Pathol*. **2018**, *13*, 84.
106. Bindhu, J.; Imtiaz, A.; Kumar, R.V.; Thejaswini, M.D.R.T. Cystic variant of favorable-histology Wilms' tumor presenting with osteolytic metastasis to the ribs. *J. Postgrad. Med.* **2010**, *56*, 28–30.

107. Jenkins, M.C.; Allibone, E.B.; Berry, P.J. Neuroglial tissue in partially cystic Wilms' tumour. *Histopathology* **1991**, *18*, 309–313.
108. Reinberg, Y., Anderson, G.F.; Franciosi, R.; Manivel, C.; Dehner, L.P.; Burke, B.A. Wilms tumor and the VATER association. *J Urol.* **1988**, *140*, 787–789.
109. Wood, B.P.; Muurahainnen, N.; Anderson, V.M.; Ettinger, L.J. Multicystic nephroblastoma: Ultrasound diagnosis (with a pathologic-anatomic commentary). *Pediatr. Radiol.* **1982**, *12*, 43–46.
110. Datnow, B.; Daniel, W.W.J. Polycystic nephroblastoma. *JAMA* **1976**, *236*, 2528–2529.
111. Ariel, I.; Abeliovich, D.; Bar-ziv J.; Hochberg, A. Renal pathology in WAGR syndrome. *Pediatr. Pathol. Lab. Med.* **1996**, *16*, 1013–1021.
112. Nakamura, Y.; Nakashima, T.; Nakashima, H.; Hashimoto, T. Bilateral cystic nephroblastomas and botryoid sarcoma involving vagina and urinary bladder in a child with microcephaly, arhinencephaly, and bilateral cataracts. *Cancer* **1981**, *48*, 1012–1015.
113. Keegan, G.T.; Peterson, R.F.; Stucki, W.J.; Street, L. Case report: Cystic partially differentiated nephroblastoma (Wilms tumor). *J. Urol*. **1979**, *121*, 362–364.
114. Nakamura, Y.; Nakashima, H.; Fukuda, S.; Hashimoto, T.; Maruyama, M. Bilateral cystic nephroblastomas and multiple malformations with trisomy 8 mosaicism. *Hum Pathol.* **1985**, *16*, 754–756.
